# Lateralized and bilateral sensory mechanisms independently regulate sociability and social preference in *Drosophila*

**DOI:** 10.3389/fnbeh.2026.1745784

**Published:** 2026-07-06

**Authors:** Chi-Lien Yang, Ching-Hsin Chen, Ching-Che Charng, Kuan-Lin Feng, Hung-Yin Tsai, Ann-Shyn Chiang

**Affiliations:** 1 Institute of Systems Neuroscience, National Tsing Hua University, Hsinchu, Taiwan; 2 Brain Research Center, National Tsing Hua University, Hsinchu, Taiwan; 3 Department of Power Mechanical Engineering, National Tsing Hua University, Hsinchu, Taiwan; 4 Institute of Physics, Academia Sinica, Taipei, Taiwan; 5 Department of Biomedical Science and Environmental Biology, Kaohsiung Medical University, Kaohsiung, Taiwan; 6 Institute of Molecular and Genomic Medicine, National Health Research Institutes, Zhunan, Miaoli, Taiwan; 7 Graduate Institute of Clinical Medical Science, China Medical University, Taichung, Taiwan

**Keywords:** lateralization, sensory integration, sociability, social behaviors, social preference

## Abstract

Social behavior involves sociability—the drive to interact—and social preference—the choice of partners. Using *FlySocialer*, an automated system for tracking group interactions, we analyzed male *Drosophila melanogaster* and found highly individualized, non-random social behaviors. Compared to randomized controls, flies showed stable individual differences in sociability and distinct partner preferences. Sociability exhibited a sensory-specific lateralized asymmetry: visual inputs via the left eye modulated interaction number, while olfactory inputs via the left antenna influenced interaction duration. Conversely, neither sensory manipulation exerted a significant effect on social preference. These results reveal distinct mechanisms for sociability and selectivity, highlighting lateralized sensory drive versus bilateral integration as core principles of social behavior in *Drosophila*.

## Introduction

Brain lateralization refers to the functional specialization of neural circuits across the left or right hemispheres, which asymmetrically govern distinct cognitive, sensory, and motor processes (Rogers, 2009; Cavelius et al., 2022). Rather than processing information symmetrically, lateralized brains often exhibit sensory asymmetries where one hemisphere or unilateral sensory organ takes dominance over specific environmental cues. For instance, humans exhibit hemispheric asymmetry in emotional processing (Ahern and Schwartz, 1985; Fusar-Poli et al., 2009; Palomero-Gallagher and Amunts, 2022). And the emotional valence can be modulated by social learning (Shamay-Tsoory et al., 2008). Also, other mammals like cats use their right nostril when investigating the odor of unknown humans (Miyairi et al., 2025). Similarly, invertebrates like honeybees rely on the right antenna to process context-appropriate social cues during dyadic interactions (Rogers et al., 2013), demonstrating that sensory lateralization also extends directly to learning and memory formation (Letzkus et al., 2008; Anfora et al., 2010; Frasnelli et al., 2010, 2012). Despite this evidence, the specific neural circuits governing sensory lateralization during social interactions in invertebrates remain largely unknown.

*Drosophila melanogaster* (the fruit fly) has emerged as a powerful model organism for dissecting the neural basis of such complex behaviors. A key advantage of *Drosophila* is its fundamental similarity in genetic and biochemical pathways shared with mammals, despite anatomical differences (Pandey and Nichols, 2011; Verheyen, 2022). Furthermore, *Drosophila* exhibits a rich repertoire of social behaviors, such as courtship, aggression, and group interaction. This model also possesses an advanced genetic toolkit and a well-characterized neurobiology, which allow for precise manipulation of neural circuits (Sethi and Wang, 2017; Garcia-Marques et al., 2019; Luan et al., 2020).

Among these lateralized behaviors, social interaction is exceptionally complex, requiring the brain to integrate multi-modal sensory cues from conspecifics. These interactions reveal two distinct dimensions: (i) sociability, defined as the baseline motivation to initiate and maintain social contact; (ii) social preference (or selectivity), which governs choice-directed behavior toward specific conspecifics based on social valence. Although lateralized sensory processing has been linked to general social contexts, whether and how unilateral sensory inputs independently modulate the absolute drive of sociability versus the discriminatory cognitive processing of social preference remains poorly understood.

To leverage the advantages of *Drosophila* in studying the neural circuitry and molecular mechanisms underlying social behavior, a well-designed behavioral assay capable of analyzing interactions among multiple individuals within large populations is required. In this study, we developed *FlySocialer*, an automated behavioral monitoring system that can simultaneously track the locomotion and social interactions of more than 20 flies. By incorporating the medial axis of overlapping body regions and wing position information, we greatly improved segmentation accuracy under crowded conditions. In addition, pigment labeling enhanced the stability of individual identity tracking. Using this system, we found that vision alone significantly influences the initiation of social interactions, whereas olfaction alone is essential for sustaining them. Interestingly, sensory inputs—both visual and olfactory—from the left side play a more prominent role in social behavior, providing the first evidence for lateralization of social behavior in *Drosophila*.

## Materials and methods

### Experimental setup

For our system, we utilized the VH-4MC-M/C 20 camera (Vieworks, Anyang, South Korea) paired with the FV3526L-F lens (Myutron, Tokyo, Japan). The camera provides a resolution of 2048 × 2048 pixels and operates at a frame rate of 20 fps. The lens features a 35 mm focal length and a resolution of 19.5 pixels/mm. Detailed specifications are available in Table 1.

**TABLE 1:** Camera and lens specifications.

Parameter	Specification
Camera model	VH-4MC-M/C 20
Resolution	2,048 × 2,048
Frame rate	20 fps
Optical format	4/3”
Sensor model	KAI-4021
Pixel size	7.4 μm × 7.4 μm
Lens model	FV3526L-F
Focal length	35 mm
Working distance	180 mm
Magnification rate	0.2
Resolution	19.5 pixels/mm

### FlySocialer analysis software

*FlySocialer* is designed to analyze the locomotion and social interactions of fruit flies. Detailed information about the software is provided in Supplementary material. The software is executed in MATLAB, and the source code is freely available on GitHub.^1^

### Fly stock

Fly stocks were maintained on cornmeal food at 22°C and 70% relative humidity with a 12:12 h light/dark cycle. The following strains were used: *Canton-S* (BDSC 64349), *UAS-shibire^ts^* (BDSC 44222), *Orco-Gal4* (BDSC 23292), *w*^1118^ mutant (BDSC 3605), and *Orco*^1^ mutant (BDSC 23129). *GMR-Gal4* is a gift from Dr. Tzu-Kang Sang. For group-rearing and socially isolated, male flies were collected 3 hours after eclosion. In group-rearing, 10–15 male flies were housed in a vial with standard food for at least 5 days post-eclosion. In socially isolated, individual males were kept in separate vials for the same duration. All of the experiments in this study were done with flies aged 3–10 days after eclosion. The fly stocks used in this study were approved by the Ministry of Agriculture, Executive Yuan, Taiwan. All experiments were conducted at National Tsing Hua University (NTHU). No private or protected land was accessed, and no protected species were sampled. After the experiments, all flies were euthanized by freezing at −20°C.

### Fly coding

One day before the experiments, flies were labeled with acrylic paint on the dorsal thorax using binary color codes: red + red, red + blue, red + green, blue + red, blue + blue, blue + green, green + red, green + blue, green + green, and gold + gold.

### Eye-blocking and antennectomy

All procedures were performed on flies seven days post-eclosion. Flies were divided into two separate experimental cohorts. For eye-blocking procedure, eyes were painted with black acrylic paint. Any individuals with paint covering other body parts were discarded. For antennectomy procedure, antennae were surgically removed using micro dissecting forceps. Following either procedure, all flies were allowed a 1-day or 4-days recovery period before subsequent behavioral experiments.

### Behavioral assay

**FIGURE 1:**
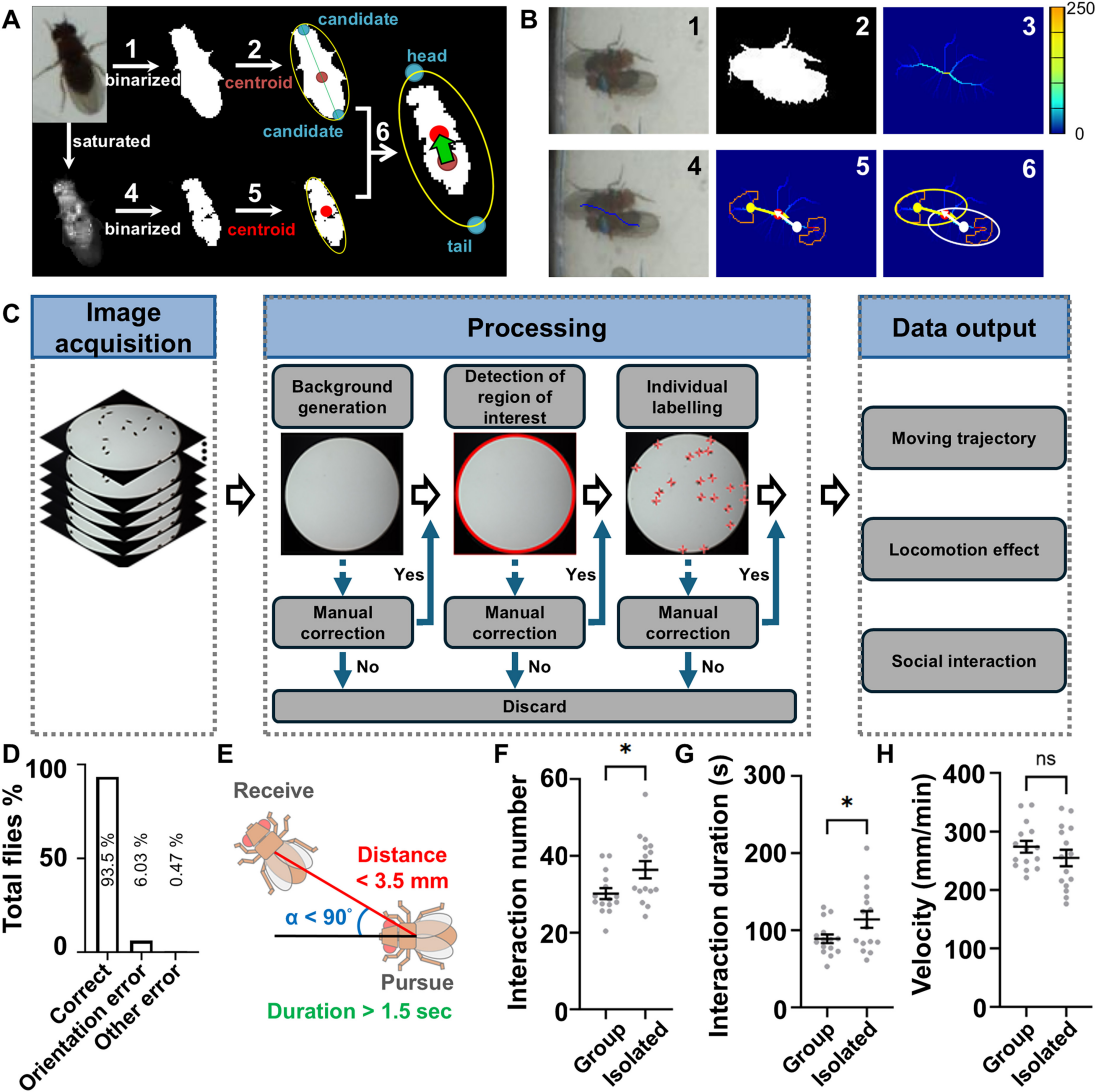
The *FlySocialer* tracking system. **(A)** Orientation detection. The head direction of each fly is defined by the vector from the centroid of the raw image (brown) to that of the saturated image (red). **(B)** Separation of overlapping flies. (1) Raw image of multiple flies. (2) Binary mask showing overlapping regions. (3) Distance map indicating medial axis candidates (color scale, 0–250; 0 = boundary, 250 = geometric center). (4) Medial axis (blue) and key points: highest probability (red) and axis termini (yellow, white). (5) Head estimation by extending the major axis toward the centroid. (6) Predicted ellipses (yellow, white) aligned with wing edges (orange). **(C)** Workflow of fly extraction. Image subtraction identifies approximate fly positions, followed by background modeling, ROI detection, and labeling to define individual coordinates for trajectory and social analyses. **(D)** Error rate of the *FlySocialer* system. Quantification of the system’s tracking accuracy and error profiles, consisting of correct tracking, orientation error, and other error (which combines detection and merge-split errors) validated against 1,277 manually annotated flies as ground truth. **(E)** Definition of social interaction. **(F–H)** Behavioral differences between group-reared and socially isolated *Canton-S* males. **(F)** Interaction number (*p* = 0.0269), **(G)** interaction duration (*p* = 0.0499), and **(H)** velocity (*p* = 0.2770). Socially isolated males exhibited a higher interaction number and longer interaction durations, whereas their locomotor velocity remained comparable to that of group-reared males. Data are presented as mean ± SEM (*n* = 15 independent experiments, with a total of 150 flies). Statistical significance was determined by unpaired *t*-test, *p < 0.05.

The experimental arena consisted of a circular plastic plate (50 mm diameter, 3 mm height) with a glass roof. The wall of the plastic plate was coated with talcum powder (Ning et al., 2019), and the glass roof was treated with a water repellent to prevent fruit flies from climbing. Experiments were conducted between 10:00 and 18:00. For the validation experiments involving group-reared and socially isolated flies (Figure 1), ten male flies (3–5 days post-eclosion) were introduced into the arena without CO_2_ anesthetization. These assays were conducted at 20°C with external cooling fans installed to enhance heat dissipation around the arena and prevent local heat accumulation from the light source. Additionally, these flies underwent a standardized drying step (by adding dry tissue paper into the food vials) prior to the assay. For this specific validation, social metrics were calculated based on a single 10-min recording session.

For genetic and physical deprivation assays, groups of 10 male flies were briefly anesthetized with CO_2_, introduced into the behavioral arena, and allowed to acclimate for 10 min prior to recording. Each assay comprised 5 trials of 10 min each, with a 1-min recovery period after being shaken by a Vortex-Genie 2 Shaker (Scientific Industries, New York, United States) at the 3rd gear for 3 seconds. All experiments were done in recorded using the VH-4MC-M/C 20 camera (Vieworks, Anyang, South Korea) in a controlled environment maintained at 20–25°C for most experiments except 30°C for shibire experiment, and 40–50% relative humidity.

### Social interaction

Social interaction was defined based on two conditions: (1) the distance between the centroids of two flies was less than 3.5 mm (approximately the body length of a fly); and (2) this distance was maintained for over 1.5 s. The angle between the head direction line and the line connecting their centers of mass (angle α) determined the interaction type. If α was less than 90°, the interaction was categorized as active; if α was greater than or equal to 90°, it was categorized as passive.

### Simulated virtual fly

To assess the individuality of interactions, we developed a simulated fly experiment. In this setup, the initial positions of the 10 virtual flies were generated using complete spatial randomization within the predefined coordinates of the behavioral arena. The movement of each virtual fly was simulated using a discrete decision-making framework to ensure biological plausibility and kinematic smoothness. Specifically, every 4–10 frames (approximately 0.2–0.5 s), each fly independently executed a decision-making process to determine its subsequent behavior. During each decision event, movement velocity was randomly sampled from an empirical dataset derived from real Canton-S male flies recorded under identical experimental conditions (i.e., recording duration and group size). To preserve realistic acceleration and avoid abrupt kinematic transitions, the change in velocity between consecutive decisions was constrained to a maximum of 10 pixels. The heading direction for the subsequent interval was determined by randomly sampling an angular displacement within ± 30° (precision to four decimal places), calculated relative to the fly’s head direction in the immediately preceding frame. This locally constrained angular update reproduces the statistical properties of real fly locomotion and steering dynamics while explicitly excluding any form of social interaction.

### Interaction preference calculation

For each experiment, we iterated over every individual as the **focal** i and formed the vector c_*ij*_ of interactions with all other group members *j* (definition of the set of partners depends on normalization mode below). To reduce zero-count bias, we applied Laplace smoothing with α = 0.001:


$$c_{i\,j}=c_{i\,j\,\_\,r\,a\,w}+\alpha ,$$

$$p_{i\,j}=\frac{c_{i\,j}}{\sum_{k}c_{i\,k}}$$

The raw Shannon entropy was


$$H_{r\,a\,w}\,\left(p_{i}\right)=-\sum_{j}p_{i\,j}\,\ln p_{i\,j},$$

$$H_{n\,o\,r\,m}=\frac{H_{r\,a\,w}}{\ln N},0\leq H_{n\,o\,r\,m}\leq 1$$

We normalized by the log of the support size N to obtain

and defined the Preference Index (PI)


$$P\,I=1-H_{n\,o\,r\,m}$$

which increases as interactions concentrate on fewer partners.

To fully capture the nuances of social preference, the preference index was calculated using two distinct behavioral variables. First, the interaction number-based preference index quantifies initiation selectivity, reflecting the degree to which a focal fly repeatedly chooses to initiate social contact with a specific subset of preferred partners. A high value indicates a strong bias in partner choice during social approaches, whereas a low value suggests that initiations are distributed uniformly across all individuals in the arena. Second, the interaction duration-based preference index quantifies maintenance selectivity, reflecting the degree to which a fly repeatedly chooses to sustain or prolong its social encounters with those specific partners. Distinguishing between these two metrics allows us to separate a fly’s drive to seek out specific individuals from its propensity to maintain prolonged social engagement with them.

### Statistics

Statistical analyses were conducted using Prism 10 (Dotmatics, Boston, Massachusetts, United States). Each experiment was replicated at least 9 times. Results are presented as mean ± SEM (standard error of the mean). Data normality was first assessed using the Kolmogorov-Smirnov test. For data that passed the normality test, differences were assessed using two-sided Student’s *t*-test (for two groups), one-way ANOVA (for multiple groups), or two-way permutation test (for 2 × 2 factorial design in sensory deprivation assays). Within this permutation framework, the “left effect” and “right effect” evaluated the primary main effects of left-sided and right-sided sensory deprivation (eye painting or antennectomy), respectively, while the “statistical interaction effect” assessed whether the behavioral impact of manipulating one side depended on the status of the contralateral side. For data that did not pass the normality test, differences were assessed using the Mann-Whitney test (for two groups) or Kruskal-Wallis test (for multiple groups). Significance levels were defined as *P < 0.05, **P < 0.01, ***P < 0.001, and ****P < 0.0001.

## Results

### Development of *FlySocialer* for automated tracking of fruit fly social interactions

Automated behavioral tracking plays a pivotal role in quantifying high-throughput animal interactions. However, many existing tracking platforms still suffer from notable technical limitations, particularly frequent orientation errors and identity swaps during periods of close body overlapping. To overcome these limitations, we developed *FlySocialer* to establish a highly streamlined, open-architecture tracking platform designed for the precise and continuous monitoring of *Drosophila* social behavior. Groups of ten wild-type males were recorded in a circular arena (5 cm in diameter, 0.3 cm in height), and custom algorithms were implemented to improve individual identification by resolving orientation ambiguity and body overlap.

To determine head orientation (Figure 1A), the system employs the HSV (hue, saturation, value) color model to isolate transparent wings. Because wings are positioned opposite the head, the vector from the centroid of the raw image to that of the wingless (saturated) image accurately indicates head direction. To separate overlapping individuals (Figure 1B), images were binarized, and a medial-axis algorithm was applied to fit distinct ellipses to each fly. An automated module further identified regions of interest (ROI) and extracted behavioral metrics from background-subtracted images (Figure 1C). This algorithm achieved an identification accuracy of 93.5% (1,194 out of 1,277 flies identified perfectly). Detailed error analysis revealed the orientation error rate (primarily due to head-to-tail inversions or head-to-wings misclassifications) was 6.03%. We observed an exceptionally low detection error rate of 0.078%, representing instances where the system completely failed to locate an individual or falsely designated background noise as a fly. Finally, the merge-split error rate, where the head or body coordinates of adjacent flies were misassigned, was merely 0.392% (Figure 1D and Supplementary Figure 1).

### Software validation using group-reared and socially isolated flies

To validate *FlySocialer*, we compared social interaction behaviors between group-reared and socially isolated *Drosophila* (*Canton-S* males). The definition of a social interaction event is a centroid distance less than 3.5 mm and maintaining for over 1.5 s (Figure 1E).

Sociability was quantified using two metrics: interaction number and total interaction duration. Here, socially isolated flies exhibited significantly more interactions (Figure 1F), as well as longer total interaction durations than group-reared flies (Figure 1G). Notably, this heightened sociability occurred in the absence of any confounding differences in general locomotion, as both groups displayed comparable average velocities (Figure 1H). Consistently, socially isolated flies also displayed a shorter mean Euclidean distance toward other individuals, which is indicative of a heightened tendency for social clustering (Supplementary Figure 2A). These results are consistent with previous findings (Bentzur et al., 2021), validating *FlySocialer*’s capacity to accurately detect established behavioral phenotypes. Intriguingly, despite the identical group-level average velocity, individual locomotor speed was consistently negatively correlated with both interaction number and duration across both rearing conditions (Supplementary Figures 2B–E).

### Individualized social behavior

**FIGURE 2:**
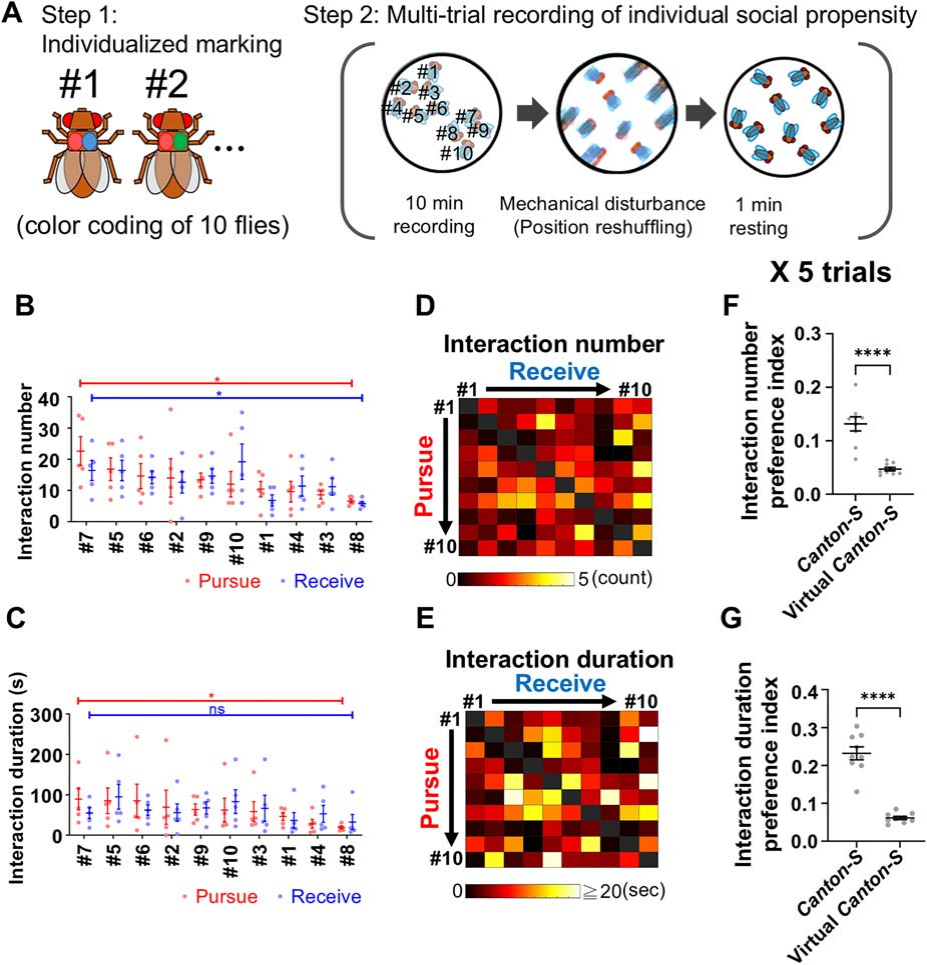
Diverse sociability and preference in wild-type males. **(A)** Experimental workflow: 10 males were color-coded for individual identification. Flies were introduced into the arena for a 10-min recording session. After each session, the arena was subjected to a mechanical disturbance to reshuffle fly positions, followed by a 1-min rest period before the subsequent trial. This procedure was repeated for 5 trials to generate social interaction movies for consistency analysis. **(B–E)** Representative data from a single independent experiment (out of the 9 total experiments, comprising 5 trials) demonstrating diverse sociability and partner preferences among the 10 individuals. **(B,C)** Interaction profiles (categorized by pursuing and receiving behaviors) displaying total interaction number and duration for individual *Canton-S* males. The highly interactive individual (#7) exhibited significantly higher interaction number and durations compared to the most solitary individual (#8). Data are presented as mean ± SEM (*n* = 5 trials). Statistical significance was determined by paired *t*-test: **(B)** pursue: *p* = 0.0253, receive: *p* = 0.0174 **(C)** pursue: *p* = 0.0432, receive: *p* = 0.1178. **(D,E)** Heatmaps of interaction number and durations among 10 real males. Social preference was quantified as a preference index (1 – normalized Shannon entropy), with higher values indicating stronger partner bias. Complete datasets for all other 8 independent experiments are presented in Supplementary Figure 5. **(F,G)** Real males showed significantly higher preference indices based on both interaction number and durations than virtual males. Data are presented as mean SEM (*n = 9* independent experiments, total 90 flies). Statistical significance was determined by unpaired *t*-test: **(F)** *p* = 0.0010, **(G)** *p* = 0.0007. Significance: *P < 0.05, ****P < 0.0001.

Beyond overall sociability, we examined individualized social behaviors to determine whether flies exhibit stable, non-random interaction patterns. We quantified social preference, defined as a consistent preference for specific partners, as a salient feature of these interactions. To eliminate spatial inertia and ensure that social preferences were maintained across changing contexts, the 50-min assay was divided into five 10-min trials separated by brief mechanical reshuffling to disrupt the flies’ physical positions (Figure 2A). The re-establishment of coordinated contact with identical partners despite these randomized initial positions provides compelling evidence of active partner choice. To ensure accurate longitudinal tracking across these disconnected trials, each fly was assigned a unique combination of thoracic color-coded tags (Supplementary Figure 3A), allowing reliable identity recognition throughout the recordings. Crucially, control experiments demonstrated that this color-labeling procedure did not affect the flies’ social interactions (Supplementary Figures 3B,C). To evaluate the randomness of these behaviors, we compared empirical data against simulated “virtual flies,” which were modeled to match the velocity profiles of real flies but exhibited randomized movement trajectories (see Materials and methods).

To assess sociability, we quantified the number and total duration of interactions for each individual across 5 trials. While individual data points expectedly spanned a notable range, significant differences in both interaction number and duration were observed between the most social (Fly #7) and least social (Fly #8) individuals (Figures 2B,C). In contrast, virtual *Canton-S* flies showed uniform interaction levels, lacking such individualized behavioral signatures (Supplementary Figure 4).

We next examined social preference, or partner preference. Heatmaps revealed that real flies exhibited distinct and partner-specific interactions. Using the data from our representative experiment as an illustrative example (Figures 2D,E), Fly #7 frequently engaged with flies #3, #4, and #10 but rarely with #1 or #8. Rather than an isolated anomaly, this type of individualized, selective interaction pattern—where specific flies consistently polarize their social choices—was characteristically observed across all nine independent groups (90 flies total) (Supplementary Figure 5). Virtual flies, by contrast, exhibited little to no partner preference (Supplementary Figure 6). To better characterize the temporal dynamics of social interactions, we performed longitudinal analysis across the assay duration. We plotted the cumulative interaction number and duration for the top three most active pairs over the 10-min recording period (Supplementary Figures 7A,B). Intriguingly, the individual cumulative curves of these highly active pairs revealed that social interactions were not uniform over time but instead occurred in distinct, high-intensity temporal bursts, indicated by the punctuated steep slopes. To visualize all pairwise interactions simultaneously, we generated heatmaps showing the evolution of interaction metrics over time (Supplementary Figures 7C,D). The clear expansion of high-interaction zones (white/bright regions) as the assay progressed demonstrates that partner-specific interactions become progressively more intense and spatially clustered over time.

To evaluate the specificity of social groups, we quantified social selectivity using a preference index. This index scales maximally as an individual’s interactions concentrate onto a restricted subset of partners, whereas it minimizes as interactions become uniformly dispersed across all individuals within the group (for calculation details, see Materials and methods). Our analysis confirmed that real *Canton-S* males exhibited significantly higher social preference than virtual controls (Figures 2F,G). This pronounced divergence demonstrates that while virtual controls interact indiscriminately, wild-type *Canton-S* males display robust partner discrimination, actively directing their behavior toward specific social partners. Together, these findings demonstrate that *FlySocialer* robustly detects individualized social phenotypes, revealing that wild-type males display both heterogeneous sociability and stable partner-specific preferences.

### Lateralized inputs govern sociability, while bilateral input comparison governs social preference

To evaluate sensory dependencies in social behavior, we initially examined chronic mutants (Supplementary Figure 8) and acute genetic silencing via the temperature-sensitive *shibire^ts^* transgene (Supplementary Figure 9). Among the chronic lines, only olfactory *Orco*^1^ mutants displayed a significant reduction in social interactions despite a higher baseline velocity. This elevated locomotion was likely a product of the genetic background, which confounds the interpretation of their social phenotype. Similarly, in the acute silencing assays at the restrictive temperature (30 °C), blocking either visual (*GMR-Gal4 > UAS-shibire^ts^*) or olfactory (*Orco-Gal4 > UAS-shibire^ts^*) neurotransmission yielded no significant behavioral changes relative to temperature controls. However, thermal exposure generically elevated velocity across all genotypes, where even the *Gal4*-alone controls exhibited significant temperature-dependent variations. Given our finding that locomotor velocity is negatively correlated with social interactions (Supplementary Figures 2B–E), these temperature-induced velocity confounds made it impossible to interpret the true sensory phenotypes. Therefore, these genetic approaches remained entirely inconclusive. To bypass these constraints, we next turned to temperature-independent physical deprivation assays.

**FIGURE 3:**
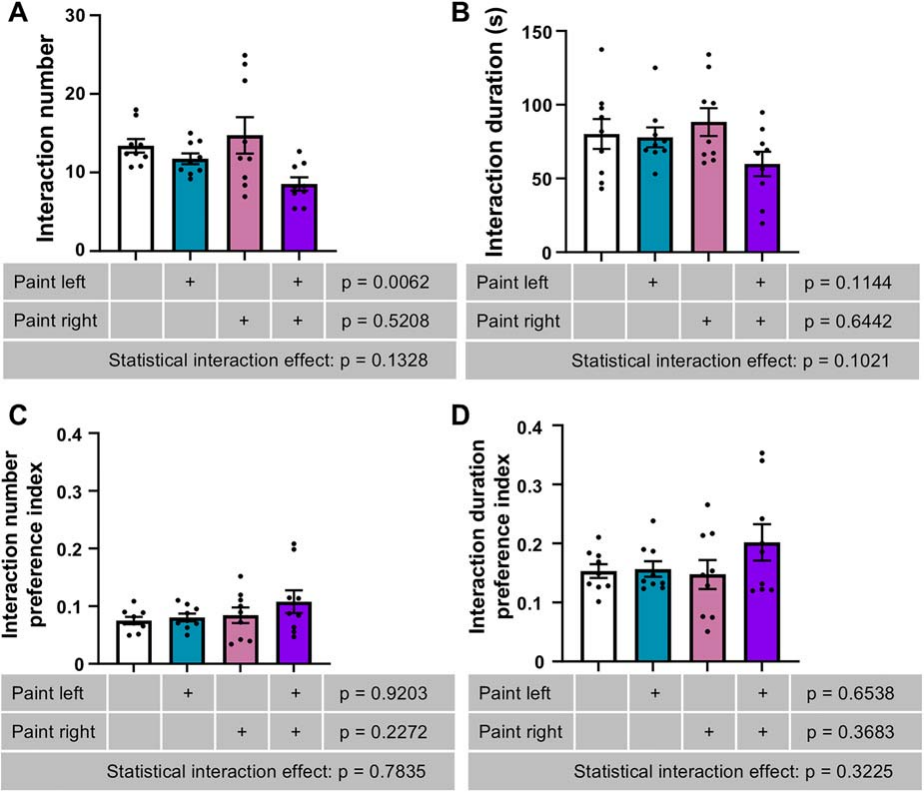
Left visual input exerts a dominant main effect on social interaction number. To investigate visual lateralization, social behavioral metrics were evaluated following unilateral visual deprivation. **(A)** Total interaction number and **(B)** total interaction duration for control, left eye painted, right eye painted, and all eyes painted *Canton-S* males. **(C,D)** Visual deprivation exerts no significant effect on social preference, as indicated by the preference indices calculated from **(C)** interaction number and **(D)** interaction duration. Data are presented as mean ± SEM (*n* = 9 independent experiments, total 90 flies). Statistical significance was determined by two-way permutation analysis (with exact *p*-values indicated in the figure).

Utilizing a two-way permutation framework within a 2 x 2 factorial design, we evaluated the independent contributions of the left and right sensory pathways. We first analyzed the effects of visual input manipulations (Figure 3). The factorial analysis revealed that visual modulation of social interaction initiation is strictly lateralized: left-eye painting exerted a significant main effect on reducing the interaction number (left effect), whereas right-eye painting caused no significant alteration (Figure 3A). Neither left- nor right-eye manipulation significantly affected the total interaction duration (Figure 3B). Furthermore, social preference indices calculated from either interaction number or duration remained entirely unaffected by unilateral or bilateral visual blockades (Figures 3C,D).

**FIGURE 4:**
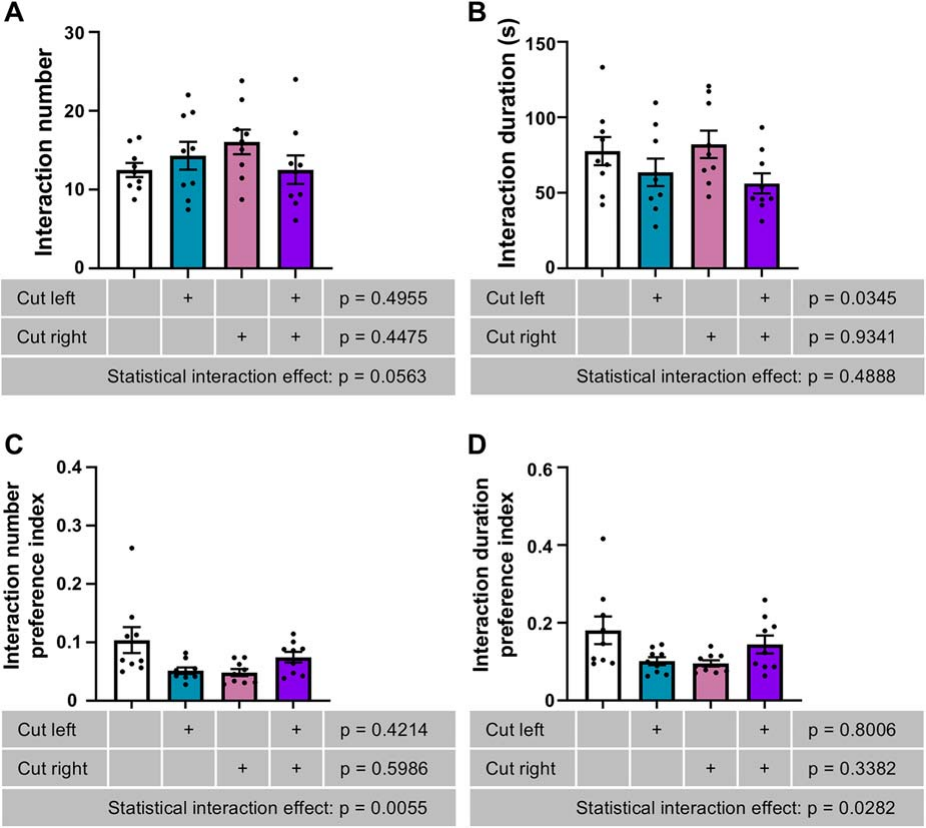
Left olfactory input exerts a dominant main effect on social interaction duration. To investigate olfactory lateralization, social behavioral metrics were evaluated following unilateral olfactory deprivation. **(A)** Total interaction number and **(B)** total interaction duration for intact, left antenna amputated, right antenna amputated, and all antennae amputated *Canton-S* males. **(C,D)** Olfactory deprivation exerts no significant effect on social preference, as indicated by the preference indices calculated from **(C)** interaction number and **(D)** interaction duration. Data are presented as mean ± SEM (*n* = 9 independent experiments, total 90 flies). Statistical significance was determined by two-way permutation analysis (with exact *p*-values indicated in the figure).

We next examined olfactory inputs using the same statistical framework (Figure 4). In contrast to the visual phenotypes, unilateral or bilateral antennectomy exerted no significant effect on the total interaction number, despite a subtle visual upward trend in the right-antenna-ablated group (Figure 4A). For interaction duration, while the four groups showed overlapping data distributions, a slight downward trend was visually apparent in the left-antenna and bilateral amputation (cut all) groups (Figure 4B). Two-way permutation test analysis revealed that interaction duration is modulated by a distinct olfactory asymmetry: losing left-side input drove a significant reduction (left effect, *p* = 0.0345), whereas losing right-side input yielded no change (right effect, *p* = 0.9341) (Figure 4B). Crucially, the statistical interaction effect was firmly non-significant for both metrics (*p* = 0.0563 for interaction number; *p* = 0.4888 for duration), verifying that left-antenna deprivation suppresses interaction duration independently of right-antenna status. Conversely, social preference indices were not significantly altered at the main effect level (Figures 4C,D). Interestingly, however, the statistical interaction effects for preference indices calculated from both interaction number (*p* = 0.0055) and duration (*p* = 0.0282) were significant. This indicates that left and right olfactory pathways functionally interact, suggesting that bilateral sensory comparison governs social preference.

Collectively, these findings suggest a sensory-specific left-hemisphere dominance in *Drosophila* social behavior. Rather than relying on a symmetric bilateral system, *Drosophila* utilize lateralized left-side inputs in a functionally segregated manner: visual inputs on the left are required to modulate interaction initiation (number), whereas olfactory inputs on the left are critical to maintain interaction persistence (duration). Conversely, although sensory lateralization appears dispensable for social selectivity at the level of individual left/right effects, the emergence of social preference relies on the functional integration of bilateral olfactory streams.

### Dynamic behavioral adjustments during chronic sensory deprivation

To test whether these sensory-driven behavioral deficits persist over time, we evaluated 4 days post-manipulation to assess potential chronic adaptation (Supplementary Figure 10). Intriguingly, long-term sensory loss induced distinct behavioral trajectories that suggest functional reorganization.

We first examined the effects of prolonged visual deprivation using flies whose eyes were covered with black acrylic paint for 4 days (Supplementary Figure 10A). Following this chronic deprivation, the acute impairments vanished entirely; social interaction metrics, including interaction number (Supplementary Figure 10B) and interaction duration (Supplementary Figure 10C), rendered statistically comparable to those of naïve controls. Furthermore, this behavioral recovery was not driven by altered locomotion, as no significant differences were observed in baseline velocity between the naïve and eye-painted groups (Supplementary Figure 10D).

Conversely, chronic olfactory deprivation via long-term bilateral antennectomy provoked a distinct compensatory response (Supplementary Figure 10E). Four days post-amputation, the total interaction number significantly increased compared to acute phenotypes (Supplementary Figure 10F), whereas the interaction duration remained the same (Supplementary Figure 10G). Similar to the visual deprivation assay, we found no significant divergence in locomotor speed between the naïve and antennectomized groups (Supplementary Figure 10H). Collectively, these observations demonstrate that the subtle acute reduction trends in interaction number (for the eye-painted group) and interaction duration (for the antenna-amputated group) do not persist chronically. Instead, the underlying neural circuitry exhibits substantial plasticity, enabling long-term behavioral compensation or sensory adaptation to overcome bilateral sensory loss.

## Discussion

### FlySocialer precisely detect social interactions of *Drosophila*

Using *FlySocialer*, an automated high-throughput behavioral tracking system developed in this study, we were able to monitor and quantify the locomotion and social interactions of more than 20 flies simultaneously with over 99.9% identity-tracking accuracy. This precision allowed robust quantification of individual and group-level variations in behavior. Our data not only corroborate previous reports on the effects of rearing environments on sociality (Bentzur et al., 2021), but also establish *FlySocialer* as a powerful platform for detecting fine-scale social individuality and dissecting its sensory and neural underpinnings.

Intriguingly, while our results align with previous studies utilizing direct spatiotemporal measurements (Bentzur et al., 2021), our finding that socially isolated flies exhibit significantly increased interaction numbers and durations appears to contrast with several landmark reports indicating reduced sociability or aggregation in isolated individuals (Simon et al., 2012; Jiang et al., 2020; Sun et al., 2020). We propose that these apparent contradictions might stem from a shift in social group-size preference in isolated flies combined with the inherent constraints of different behavioral metrics, rather than a genuine loss of sociability. Specifically, the metrics utilized for measuring global, population-wide aggregation trends in traditional social space assays (Simon et al., 2012; Jiang et al., 2020) can easily underestimate or overlook scattered, small-scale social interactions. Conversely, tethered fly paradigms rely heavily on a free-moving fly’s active motivation to approach a dense crowd of immobilized peers, making them ideal for measuring social attraction toward large groups (Sun et al., 2020). If long-term social isolation prompts flies to switch their preference from large crowds to decentralized, pairwise contacts while actively avoiding dense aggregates, their underlying sociability might be poorly captured by these large-scale frameworks. By providing a complementary event-based perspective, our system demonstrates that isolated flies retain a high capacity for social interaction, which manifests uniquely during localized contact.

### Distinct functional roles of visual and olfactory cues in social interaction

While classical paradigms indicate that *Drosophila* are initially attracted to moving targets via visual cues, full behavioral execution and conspecific recognition require integration with downstream sensory modalities, including tactile, olfactory, and gustatory inputs (Bray and Amrein, 2003; Schneider et al., 2012; Simon et al., 2012; Agrawal et al., 2014; Kohatsu and Yamamoto, 2015; Ramdya et al., 2015; Jiang et al., 2020; Rooke et al., 2020; Sun et al., 2020; Bentzur et al., 2021; Jeong et al., 2024). Our functional dissections focus on the visual and olfactory systems and reveal that these sensory inputs play distinct, complementary roles in regulating these social interactions (Figures 3, 4). Specifically, the loss of visual inputs led to a reduction in interaction number but not duration (Figures 3A,B). Conversely, olfactory deprivation selectively decreased interaction duration while leaving the total interaction number unaffected (Figures 4A,B). Taken together, these findings refine our understanding of sensory integration: while visual feedback serves as the primary gating mechanism to modulate the frequency of social contacts, olfaction is required to maintain the duration of social interactions. This sensory-specific division of labor is conceptually reminiscent of behavioral control principles documented in other complex *Drosophila* social contexts, such as courtship and aggression, where vision provides critical spatiotemporal tracking for peer detection and novel encounters, whereas chemosensory pathways function to sustain behavioral drive and proximity once contact is achieved (Krstic et al., 2009; Ramin et al., 2014). In conclusion, across diverse *Drosophila* social behaviors—encompassing courtship, aggression, and general conspecific interactions—the brain may deploy a shared sensory integration framework to orchestrate contextual behavioral decisions.

### Sensory lateralization in sociability

Beyond this functional separation, our results uncover a clear lateralization of sensory processing during social behavior. Unilateral sensory inhibition revealed a prominent left-side trend: blocking the left eye or left antenna produced greater reductions in sociability than blocking their right-side counterparts. Intriguingly, the nature of this asymmetry varied between sensory modalities: left-eye ablation primarily decreased interaction number, whereas left-antenna ablation markedly reduced interaction duration. Such lateralization parallels findings in other taxa, including vertebrates and insects, where hemispheric specialization enhances social processing efficiency (Daisley et al., 2009; Karenina et al., 2010; Rogers et al., 2013; Camerlink et al., 2018; Miyairi et al., 2025). Interestingly, the directionality of this bias contrasts with that reported in honeybees, where the right antenna dominates in social contexts (Rogers et al., 2013). This suggests that while lateralized social processing may be evolutionarily conserved, the specific hemisphere engaged can differ across species and behavioral contexts.

This broader principle of neural asymmetry implies that asymmetrical processing provides a general, conserved advantage for navigation and behavioral choice. Critically, such lateralized mechanisms can orchestrate direct behavioral responses based on asymmetric neural activation patterns. For instance, asymmetric activation within bilateral neural pathways has been elegantly demonstrated to guide directional choices and specific turning orientations in fruit flies (Sen et al., 2017). In a social context, it is highly conceivable that the lateralized sensory input processed during group interactions are integrated by central brain structures to evaluate social valence. The resulting asymmetry in central processing could then be translated into precisely oriented behavioral outputs, allowing the fly to execute specific social choices and spatial preferences. We speculate that *Drosophila* may employ hemispheric specialization to differentiate social cues of distinct valence or relevance, a hypothesis that can be tested through circuit-level and functional imaging analyses.

### Methodological discrepancies across genetic, mutational, and physical sensory blockades

Our results from transient genetic inhibition using *shibire^ts^* partially diverged from those of physical sensory ablation. In the temperature-dependent neurotransmission blockade experiments, neither visual nor olfactory inhibition led to decrease of social interaction metric—a finding that stand in contrast to our antennectomy and visual deprivation data. This discrepancy is likely reflected in methodological confounds associated with temperature manipulation. Because our *Canton-S* data showed locomotor velocity negatively correlates with social interaction measures (Supplementary Figure 3), the elevated temperature (30 °C) required for *shibire* may have also introduced physiological or behavioral variability that masked visual or olfactory effects. Similarly, behavioral tracking of constitutive sensory mutants revealed confounding baseline differences in locomotion. For instance, *Orco*^1^ mutants natively exhibited a higher baseline velocity, which likely stems from genetic background effects rather than the loss of olfactory signaling. Indeed, our preliminary experiments using sensory mutants underscore how genetic background-dependent variations in locomotor activity can complicate behavioral interpretations (Supplementary Figure 8). Notably, while recent evidence indicates that mechanical injury can reduce locomotor activity and shorten social distance (SD) in *Drosophila* (Jeong et al., 2024), our experimental design inherently provides a robust internal control to contextualize this concern. In our unilateral deprivation assays, *Canton-S* males displayed distinct behavioral profiles depending on the laterality of the manipulation (left versus right side deprivation). Because a generalized trauma or stress response would typically present as a symmetric behavioral decline regardless of injury location, these side-specific behavioral differences suggest that sensory-specific processing, rather than the surgical injury itself, remains a driver of the observed phenotypes. Additionally, sensory mutants used in preliminary experiments exhibited genetic background–dependent differences in locomotor activity (Supplementary Figure 9), further complicating interpretation. These observations underscore the need for experimental approaches that decouple temperature and genetic effects when probing sensory contributions to social behavior.

### Time-dependent behavioral compensation after sensory loss

Finally, we observed evidence of behavioral plasticity and sensory compensation following physical sensory deprivation. Acute visual or olfactory blockade (1 day) produced significant reductions in social interaction metrics (Figures 3, 4), yet these deficits disappeared by 4 days post-manipulation. Remarkably, interaction number even increased above baseline after 4 days of deprivation (Supplementary Figure 10). This recovery suggests that *Drosophila* can adapt to sensory loss by recruiting alternative modalities—such as gustatory or mechanosensory cues—to restore social function. This rapid compensation highlights the plasticity of the social behavior neural network in *Drosophila*, paralleling adaptive processes observed in other animals following sensory deprivation. Future studies combining connectomics, functional imaging, and neural perturbation will be critical for identifying the circuit-level mechanisms underlying this cross-modal compensation and for determining how multisensory integration maintains robust social behavior.

## Conclusion

Together, our findings reveal a specialized multisensory framework for *Drosophila* social behavior, in which left visual input is crucial for initiating interactions, whereas left olfactory input is essential for maintaining them. This consistent left-sided asymmetry underscores the lateralization of social behavior, which depends preferentially on information processing from unilateral left sensory organs. By developing and validating *FlySocialer*, we provide a powerful, open-access platform for high-resolution, individualized analysis of group social dynamics. This system bridges behavioral quantification with neural and genetic interrogation, enabling precise dissection of the sensory and circuit mechanisms that drive complex social interactions.

Beyond *Drosophila*, our results highlight general principles of lateralized sensory processing and cross-modal compensation, phenomena likely conserved across species. Future studies integrating functional imaging, connectomic mapping, and targeted perturbation will be crucial to elucidate how neural asymmetry and multisensory convergence together shape the computation of social decisions in the brain.

## Data Availability

The original contributions presented in this study are included in the article/Supplementary material, further inquiries can be directed to the corresponding authors.

## Footnotes

1
https://github.com/ASClabNTHU/FlySocialer.git
