# Supplementary material for: Lateralized and bilateral sensory mechanisms independently regulate sociability and social preference in Drosophila
Source: Front Behav Neurosci. 2026 Jul 6;20:1745784. doi: 10.3389/fnbeh.2026.1745784 (PMC13381832; doi:10.3389/fnbeh.2026.1745784)
Supplement: Supplementary file 1 [file Data_Sheet_1.pdf]

Supplementary Material

1 Supplementary Data

Our analysis software consisted of three parts: fly image extraction and recognition of fly body characteristics, definition of social interaction, and definition of grouping. The purpose of fly image extraction was to distinguish the location of flies in each frame and track fly identities throughout the entire experiment. The process of fly image extraction involved background generation, region of interest (ROI) labeling, and initial location labeling (Figure 1C). Further details are provided in the following paragraphs.

1.1 Software design for fly image extraction

1.1.1 Background generation and subtraction

This software generated the background by comparing the difference between each image and a generated clear background without fruit flies. With a maximum filter, this software could generate a clear background with only 12 selected frames of raw images (frames in one test were selected at intervals of 1000, a test of 10 minutes recording needs only 12 frames to create a background). In our data, it was observed that the background quality was sufficiently high to enable easy removal of moving objects and noise.

1.1.2 ROI labelling

To conserve analysis resources and avoid confusion in distinguishing flies between different arenas, we utilized an accurate region mask to label the ROIs. The location and radius of this accurate region mask were predetermined. During the analysis of image data, our software automatically generated an ROI file. Simultaneously, it annotated the ROIs with a red circle on the background for ease of inspection (Figure 1C). If the ROI is accurate, we can utilize the generated ROI file to apply the ROI position to all figures during data analysis. In rare instances, manual corrections may be necessary.

1.1.3 Initial locations labelling

In this session, individual fruit flies were identified based on their assigned color codes in the raw images, establishing a sequential order (Supplementary Figure 1). For instance, a fly marked with a color code of (red + red) was designated as #1, while one with a color code of (red + blue) was labeled as #2. Subsequently, users annotated the starting positions of fruit flies #1 to #10 in the determined order.

1.2 Software design for recognition of fly body characteristics

The main purpose of recognizing body characteristics was to identify the head of each fly and tracking. The procedure for recognition included centroid calculation, ellipse fitting, wing removal, and head detection (Supplementary Figure 11).

1.2.1 Centroid calculation

To determine the position of each fruit fly, the centroid of each region was calculated based on its spatial moments. The raw moment $M_{(i,j)}$ of an image with pixel intensities $I_{(x, y)}$ is expressed in equation (1). The coordinates of the centroid are then derived using raw moments, as shown in equation (2).

$$M_{i,j} = \sum_x \sum_y x^i y^i I(x, y) \quad (1)$$

where (x, y) is the coordinate in image space.

$$\text{centroid} = [\bar{x}, \bar{y}] = \left[\frac{M_{10}}{M_{00}}, \frac{M_{01}}{M_{00}}\right] \quad (2)$$

The positions of flies are extracted by the form of the centroids of each region. Body characteristics analysis takes charge of the remaining process.

1.2.2 Ellipse fitting

To determine the head orientation (vector), we first calculate the major and minor axes of each region. These parameters are derived from the second central moments. Equation (3) defines the central moment.

$$\text{Central moment} = \mu_{pq} = \sum_x \sum_y (x - \bar{x})^p (y - \bar{y})^q I(x, y) \quad (3)$$

A covariance matrix, built from these central moments, is provided in Equation (4).

$$\text{The covariance matrix of the region} = \begin{bmatrix} \mu'_{20} & \mu'_{11} \\ \mu'_{11} & \mu'_{20} \end{bmatrix} \quad (4)$$

where $\mu'_{20} = \frac{\mu_{20}}{\mu_{00}} = \frac{M_{20}}{M_{00}} - \bar{x}^2$

$$\mu'_{02} = \frac{\mu_{02}}{\mu_{00}} = \frac{M_{02}}{M_{00}} - \bar{y}^2$$

$$\mu'_{11} = \frac{\mu_{11}}{\mu_{00}} = \frac{M_{11}}{M_{00}} - \bar{x}\bar{y}$$

The eigenvalues of this covariance matrix are given by Equation (5).

$$\text{The eigenvalues of the covariance matrix}_{1,2} = \lambda_{1,2}$$

$$= \frac{\mu'_{20} + \mu'_{02}}{2} \pm \frac{\sqrt{4\mu'^2_{11} + (\mu'_{20} - \mu'_{02})^2}}{2} \quad (5)$$

The lengths of the major and minor axes are calculated as four times the square roots of these eigenvalues, as shown in Equation (6).

$$\text{The length of the major axis/ minor axis} = 4 \times \sqrt{\lambda_{1,2}} \quad (6)$$

Finally, the eigenvectors of the covariance matrix indicate the orientations of these axes. The orientation of the major axis, which represents the fly's orientation vector, is given by Equation (7).

$$\emptyset = \frac{1}{2}\arctan\left(\frac{2\mu'_{11}}{\mu'_{20} - \mu'_{02}}\right) \quad (7)$$

Fly positions are determined by the centroids of each detected region. Body characteristic analysis handles the remaining processing steps.

1.2.3 Wing removal

Wing removal is also the preprocessing to head point detection. In binary image, saturation value of body and wings were different (Supplementary Figure 12). Therefore, we could set a saturation threshold to remove wings but retain the body. The saturation value represents the purity level of color (R=red, G=green, B=blue). The definition of saturation value was in equation (8).

$$\text{Saturation} = \begin{cases} 0, & if\ C = 0 \\ \frac{C}{V}, & otherwise \end{cases} \quad (8)$$

Where C = maximum value of R, G, B – minimum value of R, G, B

V = maximum value of R, G, B

1.2.4 Head detection

Centroids (green point), orientation, and the major axis of a fly were subsequently used to assign candidates (blue points) for a head point and a tail point (Figure 1A). Then, calculate the centroid of the body without wings (red points). The candidate which was closer to the centroid of the body without wings will be defined as the head.

1.2.5 Tracking

The identity tracking method was based on rectilinear motion prediction. Since the period of two frames (0.1 seconds) was so short for flies, we could assume that the moving speed and the direction of a fly from frame t-1 to frame t were very close to that from frame t-2 to frame t-1. So, we could anticipate that the position $P_{anticipate}$ of the fly at time t, as equation (9) showed, where the P_{t-1} denoted the position of the fly at time t-1 and the P_{t-2} denoted the position of the fly at time t-2.

$$P_{anticipate} = P_{t-1} + (P_{t-1} - P_{t-2}) \qquad (9)$$

The identification was done by assigning the fly which was nearest to the predicted one the same identity.

2 Supplementary Figures

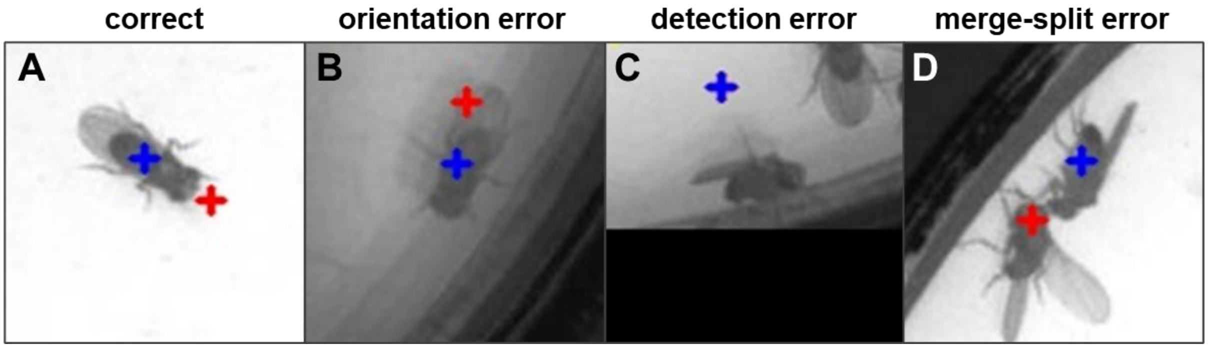

Supplementary Figure 1. Validation of FlySocialer detection. Representative images demonstrate the performance and error typologies of the tracking system: (A) correct identified, (B) orientation error, (C) detection error, and (D) merge-split error. In all panels, the blue cross indicates the calculated centroid of the fly body, and the red cross approximates the identified head position and directional alignment.

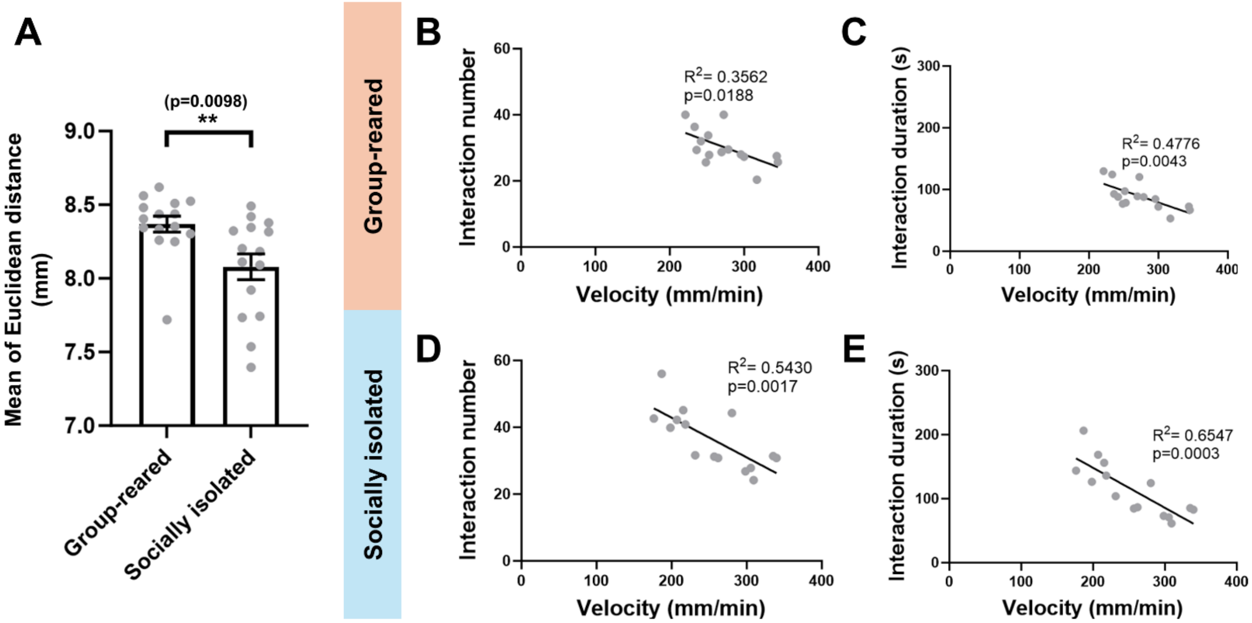

Supplementary Figure 2. Social experience alters social distance, while locomotor velocity consistently correlates negatively with social metrics in *Canton-S* males. (A) A decreased mean Euclidean distance indicates that socially isolated flies tend to display greater social clustering compared to group-reared controls. Data are presented as mean ± SEM (n = 15 independent experiments). Statistical significance was determined by Welch's t-test. Locomotor velocity of *Canton-S* males is negatively correlated with both interaction number and duration, regardless of prior social experience. (B, C) In group-reared *Canton-S* males, velocity displays a significant negative correlation with (B) interaction number and (C) interaction duration. (D, E) In socially isolated *Canton-S* males, similarly exhibits a robust negative correlation with (D) interaction number and (E) interaction duration.

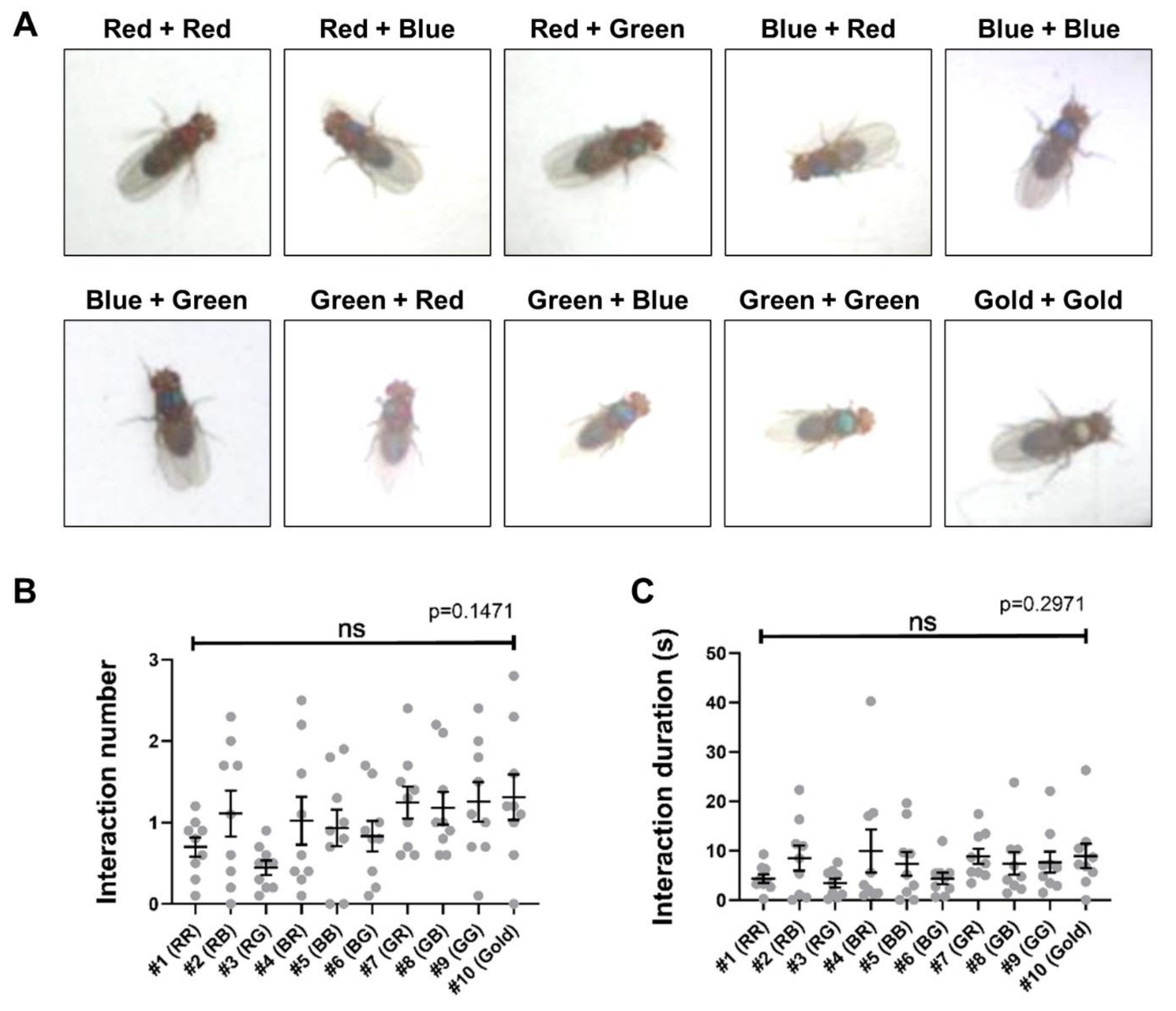

Supplementary Figure 3. Thoracic color coding facilitates robust tracking between independent experimental trials without driving social selectivity. Acrylic paint applied to the dorsal thorax of fruit flies does not act as a confounding sensory stimulus or influence social preferences. **(A)** Representative images demonstrating that the selected color codes are distinct and readily recognized by the CCD camera system; image contrast/saturation has been adjusted here solely to emphasize the spectral differences between color codes. (B, C) Quantitative behavioral validation showing that thoracic labeling exerts no significant effect on (B) interaction number or (C) interaction duration. Data are presented as mean ± SEM (n = 9 independent experiments, total 90 flies). Statistical significance was determined by one-way ANOVA.

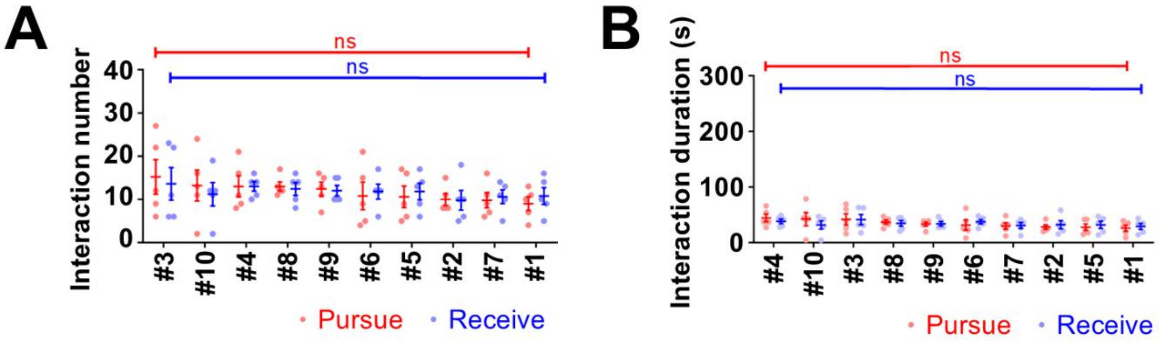

Supplementary Figure 4. Virtual *Canton-S* males with randomized social interaction partners exhibit unsegregated behavioral profiles. The virtual controls show an absence of significant behavioral segregation between the designated active and solitary cohorts across sessions. (A) Total interaction number (paired t test; pursue: $p = 0.2086$, receive: $p = 0.4597$) and (B) total interaction duration (paired t-test; pursue: $p = 0.1526$, receive: $p = 0.2742$). Data are presented as mean ± SEM ($n = 5$ trials).

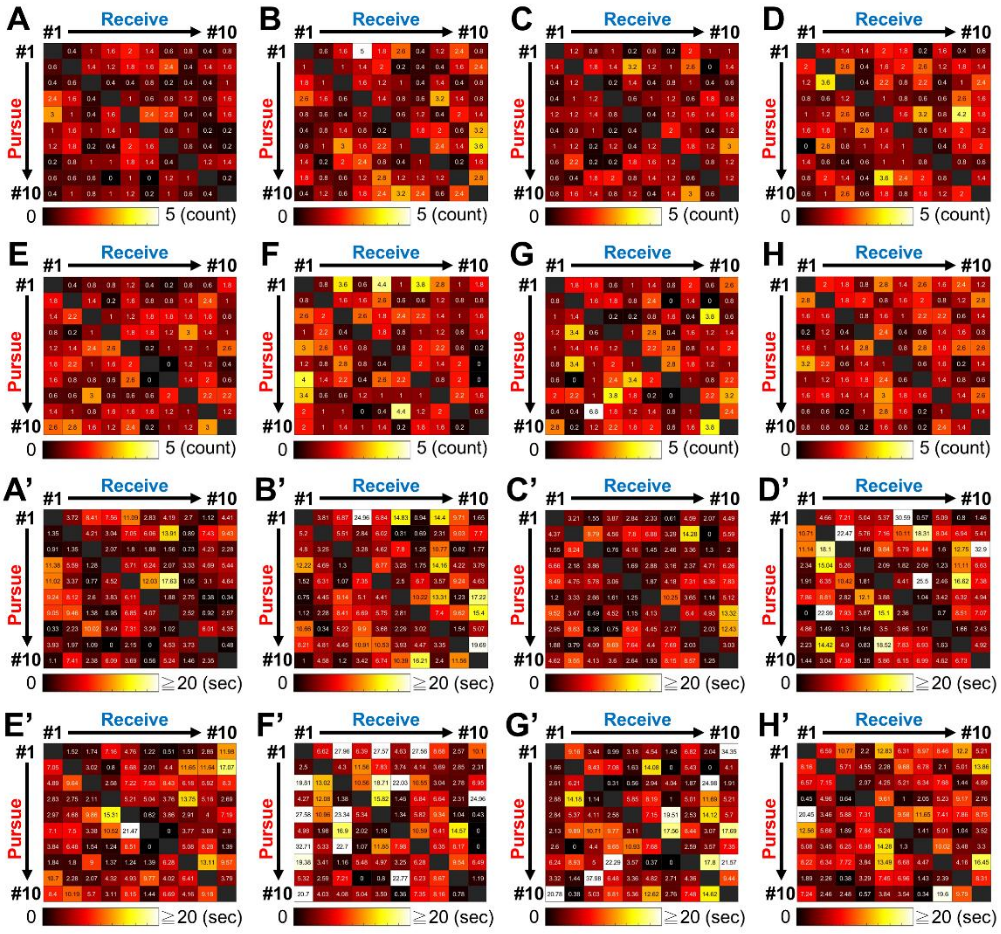

Supplementary Figure 5. Interaction number and interaction duration of *Canton-S* males. (A-H) Heat map matrix of interaction number. (A'-H') Heat map matrix of interaction duration. X and X' were from the same experiment.

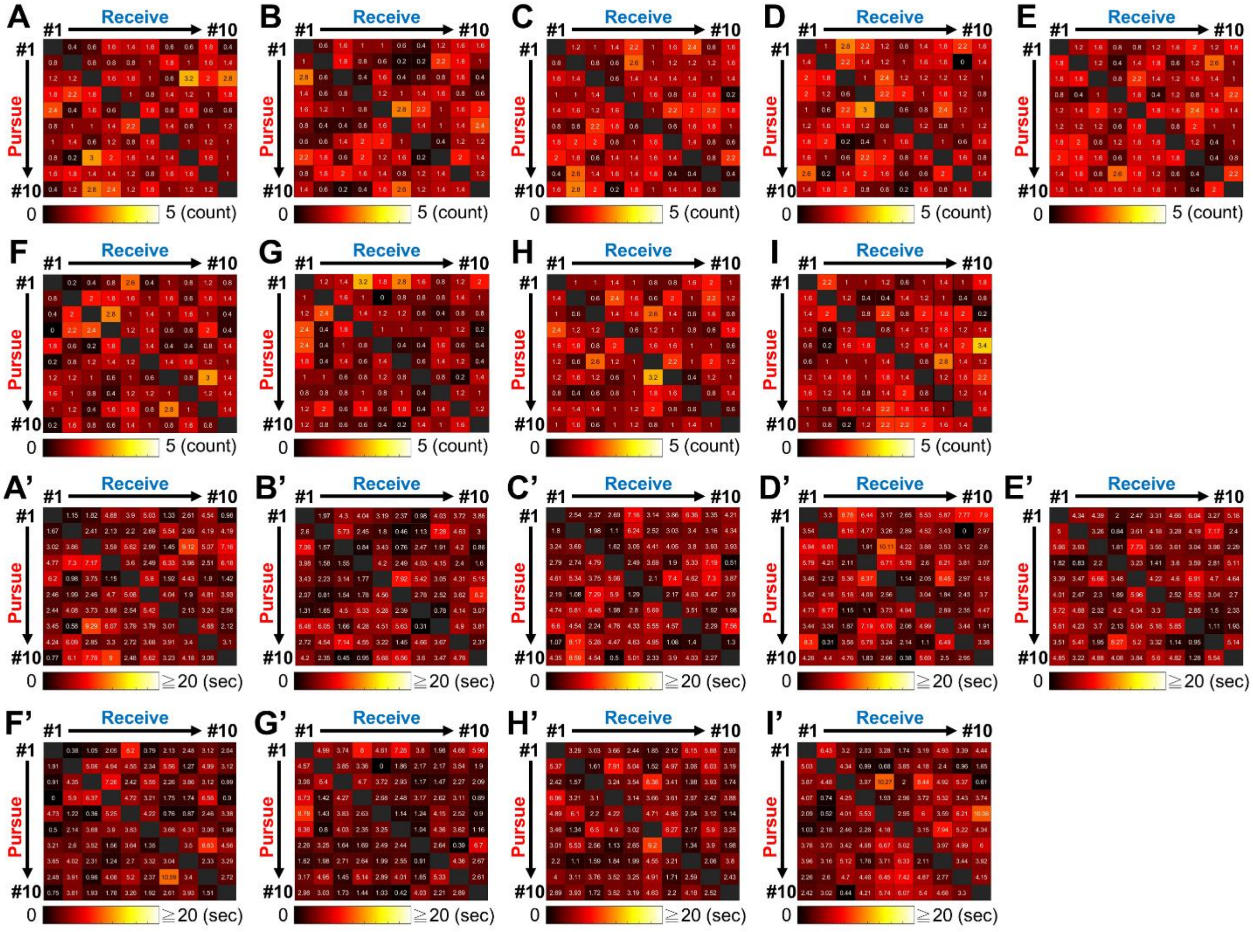

Supplementary Figure 6. Interaction number and interaction duration of virtual *Canton-S* males. (A-H) Heat map matrix of interaction number. (A'-H') Heat map matrix of interaction duration. X and X' were from the same experiment.

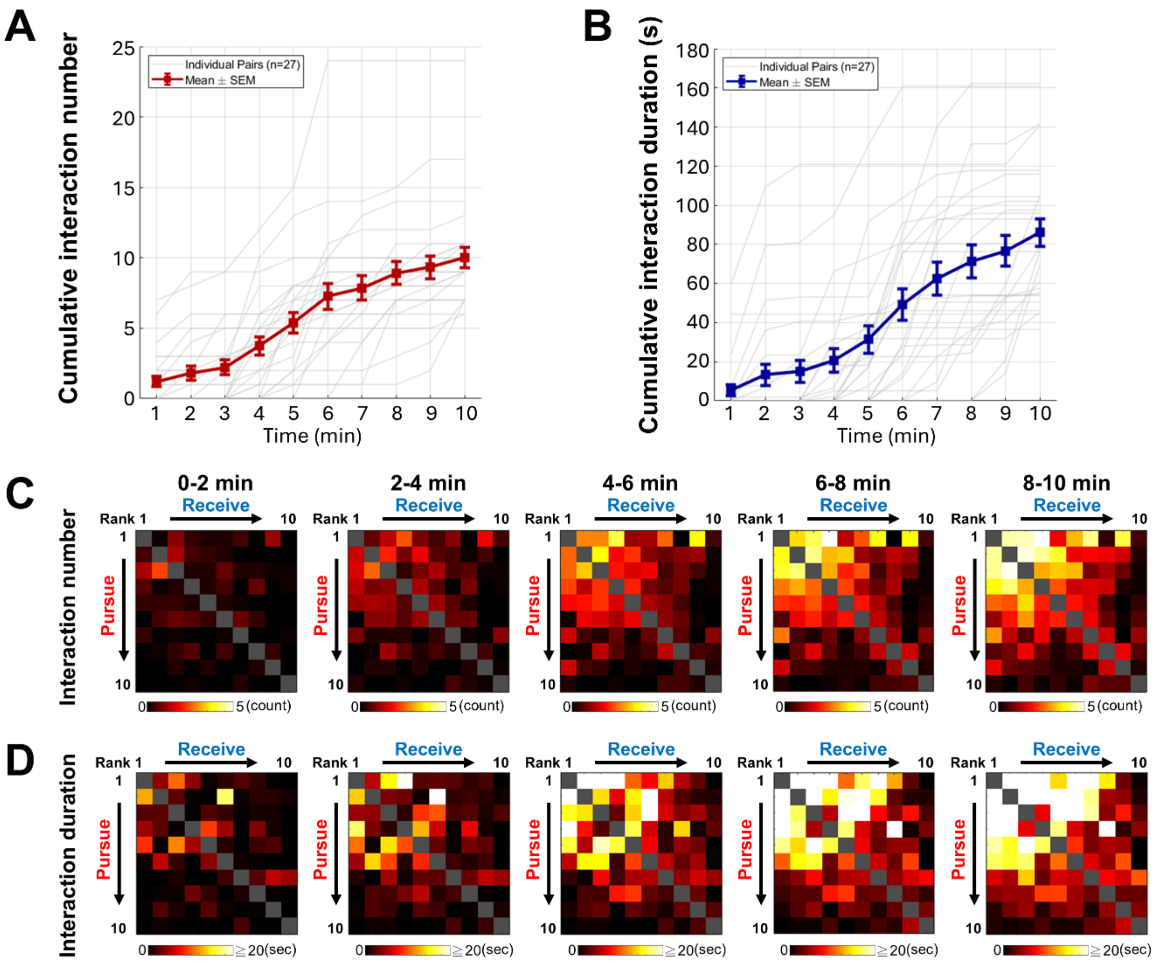

Supplementary Figure 7. Temporal bursts and dynamic intensification of partner-specific social interactions. (A, B) Cumulative interaction number (A) and duration (B) for the top three most active pairs of the first trial in 9 independent experiments over the 10-minute recording period. Darker colored lines represent the mean ± SEM, while the underlying gray curves depict the individual trajectories of all 27 highly interactive pairs. The prominent steep slopes observed within these individual gray trajectories indicate that social interactions are non-uniformly distributed, occurring instead in distinct temporal bursts. (C, D) Averaged heatmaps of all pairwise interactions for interaction number (C) and duration (D) across consecutive time windows (0-2, 2-4, 4-6, 6-8, 8-10 min). To facilitate population-level averaging, the matrix ranking was aligned based on individual interaction levels rather than arbitrary fly IDs. The progressive expansion of high-interaction zones (bright regions) over time demonstrates a significant, generalized increase in social clustering and interaction intensity as the assay proceeds.

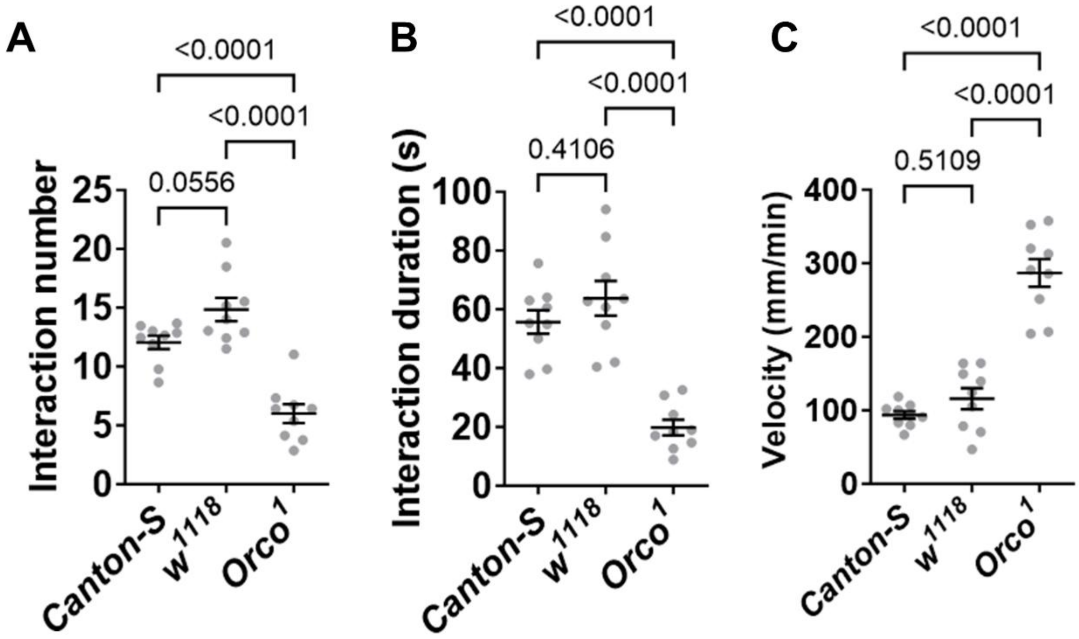

Supplementary Figure 8. Olfactory *Orco*^{1} mutants are less socially active than visual *w*^{1118} mutants and wild-type *Canton-S* flies. (A) Comparison of interaction numbers among *Canton-S*, *w*^{1118}, and *Orco*^{1} males. (B) Comparison of interaction durations among *Canton-S*, *w*^{1118}, and *Orco*^{1} males. (C) Comparison of velocities among *Canton-S*, *w*^{1118}, and *Orco*^{1} males. Data are presented as mean ± SEM (n = 9 independent experiments, total 90 flies). Statistical significance was determined by one-way ANOVA analysis (with exact p-values indicated in the figure).

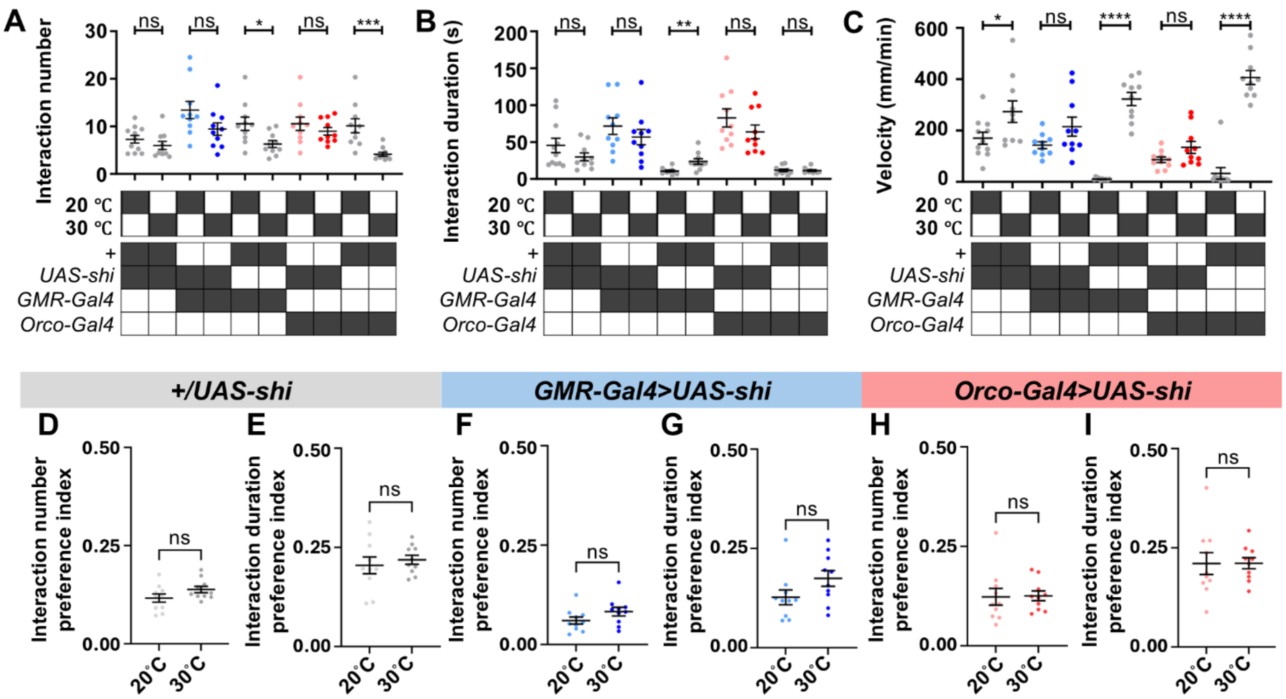

Supplementary Figure 9. Acute blockade of visual neurotransmission impairs sociability, but temperature confounds the analysis of social selectivity. (A-C) Comparison of sociability metrics and velocity across genotypes at permissive (20°C) and restrictive (30°C) temperatures. (A) Interaction number. (B) Interaction duration. (C) Velocity. Genotypes shown are control (*+/UAS-shibire*^{ts}, *GMR-Gal4/+*, *Orco-Gal4/+*), visual blockade (*GMR-Gal4>UAS-shibire*^{ts}), and olfactory blockade (*Orco-Gal4>UAS-shibire*^{ts}). (D-I) Analysis of preference index, comparing permissive (20°C) and restrictive (30°C) temperatures for each genotype. Males in each group showed no difference in preference index for both number and duration at 30°C. Data are presented as mean ± SEM (n = 10 independent experiments, total 100 flies). Statistical significance was determined as following information: for interaction number 20°C vs 30°C, p-values were as follows: *+/UAS-shibire*^{ts} (unpaired t-test, p = 0.2787), *GMR-Gal4>UAS-shibire*^{ts} (unpaired t-test, p = 0.3407), *GMR-Gal4/+* (Mann-Whitney test, p = 0.0002), *Orco-Gal4>UAS-shibire*^{ts} (Mann-Whitney test, p = 0.0753), and *Orco-Gal4/+* (unpaired t-test, p = 0.0150). For interaction duration 20°C vs 30°C, the analysis revealed: *+/UAS-shibire*^{ts} (unpaired t-test, p = 0.1885), *GMR-Gal4>UAS-shibire*^{ts} (unpaired t-test, p = 0.2376), *GMR-Gal4/+* (Mann-Whitney test, p > 0.9999), *Orco-Gal4>UAS-shibire*^{ts} (unpaired t-test, p = 0.3432), and *Orco-Gal4/+* (Mann-Whitney test, p = 0.0029). Finally, for velocity, the statistical outcomes were: *+/UAS-shibire*^{ts} (unpaired t-test, p = 0.0404), *GMR-Gal4>UAS-shibire*^{ts} (unpaired t-test, p = 0.0784), *GMR-Gal4/+* (Mann-Whitney test, p = 0.0784), *Orco-Gal4>UAS-shibire*^{ts} (unpaired t-test, p = 0.0848), and *Orco-Gal4/+* (unpaired t-test, p < 0.0001).

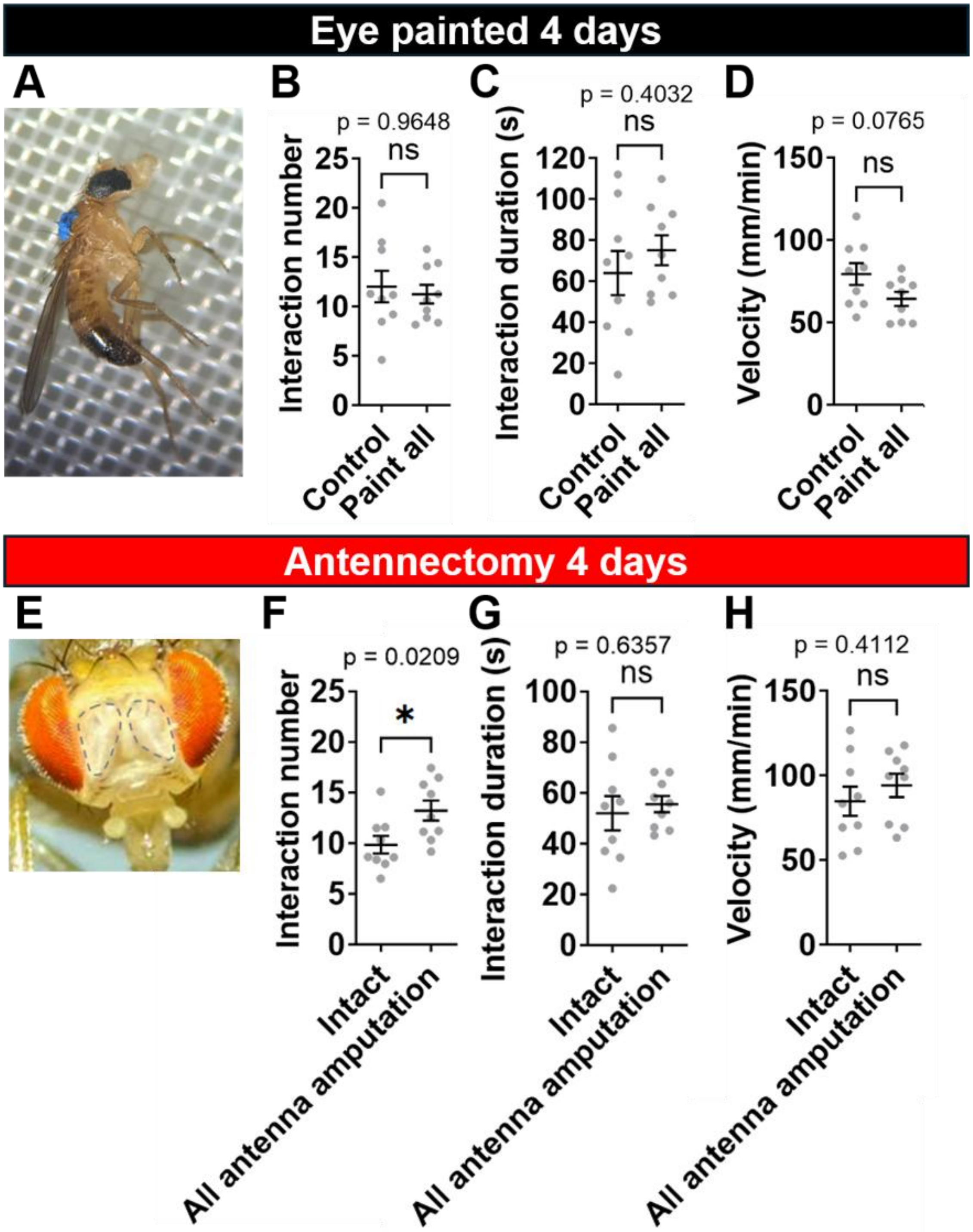

Supplementary Figure 10. Chronic sensory deprivation may induce compensatory adjustments in social behavior. (A–D) Visual deprivation assay: (A) Schematic of *Canton-S* males with eyes painted with black acrylic for 4 days, and subsequent comparisons of (B) interaction number, (C) interaction duration, and (D) locomotor velocity against naïve controls. (E–H) Olfactory deprivation assay: (E) Schematic of *Canton-S* males with antennae amputated for 4 days, alongside comparisons of (F) interaction number, (G) interaction duration, and (H) locomotor velocity against naïve controls. Data are presented as mean ± SEM (N = 9 independent cohorts per group). Statistical significance was determined by unpaired t-test, with exact p values indicated in each panel.

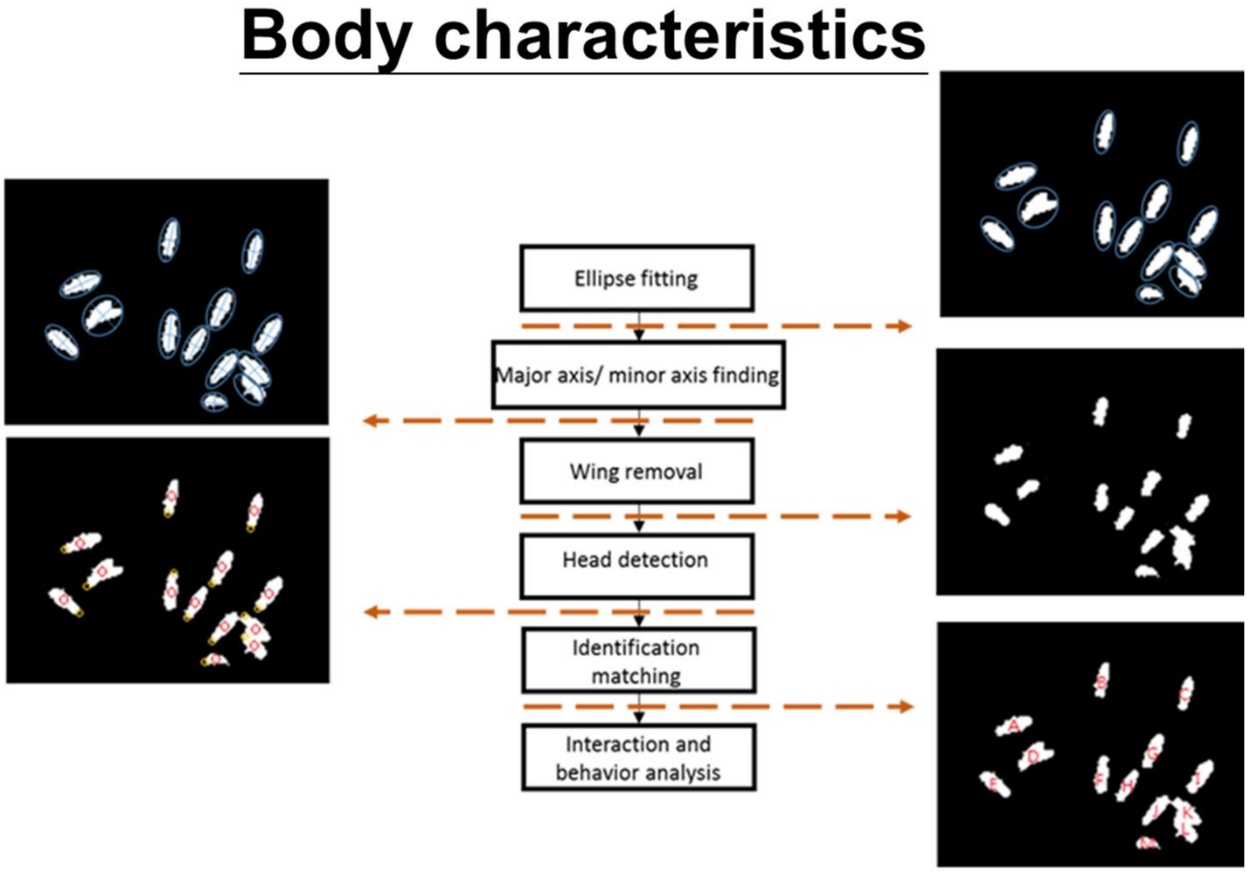

Supplementary Figure 11. The flow chart of body characteristics. The process of analyzing body characteristics includes the following steps: ellipse fitting, major axis/minor axis finding, wing removal, head detection, identification matching, interaction, and behavior analysis.

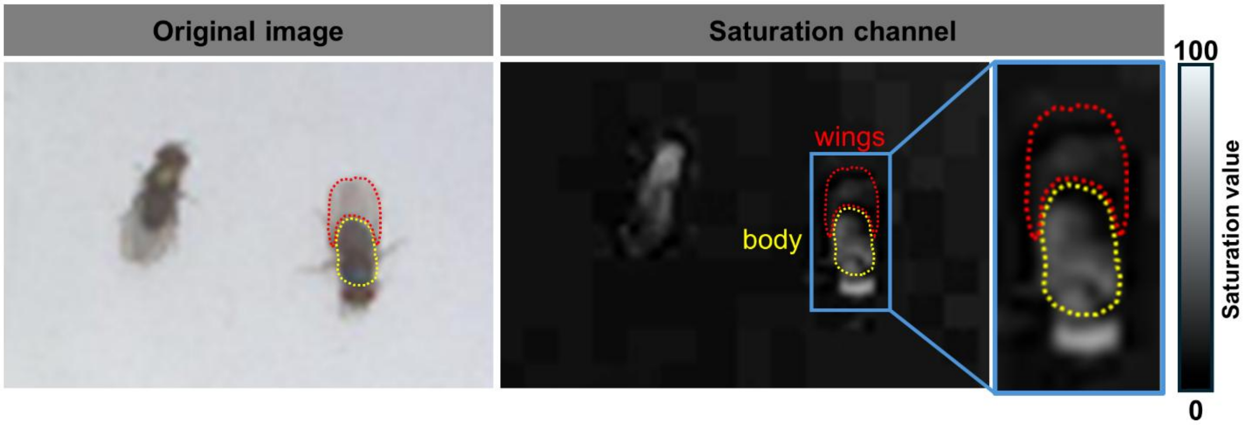

Supplementary Figure 12. The body exhibits higher saturation values than the wings. In the saturation channel, the body shows significantly higher saturation values compared to the wings, enabling clear differentiation between the two regions. Pixels with a 100% saturation value are represented in white.
